# Immune-Mediated Thrombotic Thrombocytopenic Purpura Following mRNA-Based COVID-19 Vaccine BNT162b2: Case Report and Mini-Review of the Literature

**DOI:** 10.3389/fmed.2022.890661

**Published:** 2022-05-17

**Authors:** Vanessa Alexandra Buetler, Nada Agbariah, Deborah Pia Schild, Fabian D. Liechti, Anna Wieland, Nicola Andina, Felix Hammann, Johanna A. Kremer Hovinga

**Affiliations:** ^1^Clinical Pharmacology and Toxicology, Department of General Internal Medicine, Inselspital, Bern University Hospital, University of Bern, Bern, Switzerland; ^2^Department of Hematology and Central Hematology Laboratory, Inselspital, Bern University Hospital, University of Bern, Bern, Switzerland; ^3^Department of Cardiology, Inselspital, Bern University Hospital, University of Bern, Bern, Switzerland; ^4^Department of General Internal Medicine, Inselspital, Bern University Hospital, University of Bern, Bern, Switzerland

**Keywords:** purpura, thrombotic thrombocytopenic, ADAMTS13, mRNA SARS-CoV-2 vaccine, COVID-19 vaccine

## Abstract

**Introduction:**

An increasing number of case reports have associated vaccinations against coronavirus disease 2019 (COVID-19) with immune-mediated thrombotic thrombocytopenic purpura (iTTP), a very rare but potentially life-threatening thrombotic microangiopathy, which leads to ischemic organ dysfunction. Thrombus formation in iTTP is related to a severe deficiency of the specific von Willebrand-factor-cleaving protease ADAMTS13 due to ADAMTS13 autoantibodies.

**Methods:**

We present a case of iTTP following exposure to the mRNA-based COVID-19 vaccine BNT162b2 (Comirnaty^®^, Pfizer-BioNTech). In addition, we review previously reported cases in the literature and assess current evidence.

**Results:**

Apart from our case, twenty cases of iTTP occurring after COVID-19 vaccination had been published until the end of November 2021. There were 11 male and 10 female cases; their median age at diagnosis was 50 years (range 14–84 years). Five patients (24%) had a preexisting history of iTTP. Recombinant adenoviral vector-based vaccines were involved in 19%, mRNA-based vaccines in 81%. The median onset of symptoms after vaccination was 12 days (range 5–37), with 20 cases presenting within 30 days. Treatment included therapeutic plasma exchange in all patients. Additional rituximab, caplacizumab, or both these treatments were given in 43% (9/21), 14% (3/21), and 24% (5/21) of cases, respectively. One patient died, despite a prolonged clinical course in one patient, all surviving patients were in clinical remission at the end of the observational period.

**Conclusion:**

Clinical features of iTTP following COVID-19 vaccination were in line with those of pre-pandemic iTTP. When timely initiated, an excellent response to standard treatment was seen in all cases. ADAMTS13 activity should be determined pre-vaccination in patients with a history of a previous iTTP episode. None of the reported cases met the WHO criteria for assessing an adverse event following immunization (AEFI) as a consistent causal association to immunization. Further surveillance of safety data and additional case-based assessment are needed.

## Introduction

An increasing number of case reports have associated vaccinations against coronavirus disease 2019 (COVID-19) with immune-mediated thrombotic thrombocytopenic purpura (iTTP), a very rare and potentially life-threatening form of thrombotic microangiopathy (1–3). In iTTP, autoantibodies to ADAMTS13 lead to a severe deficiency of ADAMTS13 activity (<10% of that in normal plasma) (1–3). Under physiological conditions, ADAMTS13 cleaves the ultra-large von Willebrand factor (vWF) multimers secreted by endothelial cells into smaller, less sticky vWF units. In the absence of ADAMTS13, ultra-large vWF multimers persist and bind platelets spontaneously, leading to microvascular thrombosis with variable degrees of ischemic organ damage, consumptive thrombocytopenia, and microangiopathic hemolytic anemia due to fragmentation of red blood cells as evidenced by schistocytes on the peripheral blood smear (1–3). Therapeutic plasma exchange (TPE) with replacement of plasma (4), together with immunosuppressants (corticosteroids, and often rituximab), have reduced mortality from >90% to <5% in centers experienced in the care of iTTP patients. With the addition of the anti-vWF A1 domain nanobody caplacizumab, recently available in many countries, mortality rates seem to decline further. Relapses, however, remain an issue and occur in at least 40–50% of survivors of an initial iTTP episode (1–3). With regular ADAMTS13 activity measurements at hand, survivors are nowadays followed up and may benefit from preemptive immunosuppressive therapy upon the reappearance of anti-ADAMTS13 antibodies and ADAMTS13 deficiency (5).

iTTP mainly affects adults, with peak incidences in the 3rd-5th decades in Europe and the United States (6–9). Like other autoimmune diseases, black (African-American and African-Caribbean) individuals and women are more frequently affected than non-black individuals and men. The female to male ratio ranges from 2.5–3.5 to 1. Other autoimmune disorders may be present in iTTP patients, with connective tissue disease and systemic lupus erythematosus frequently observed (10, 11).

Drug-induced acute thrombotic microangiopathies are seldom associated with a severe ADAMTS13 deficiency. Exceptions are ticlopidine, but not other members of the thienopyridine family, and drugs with immune-modulatory capacity such as revlimid and checkpoint inhibitors (12–15). More recently, several first episodes, as well as relapses of iTTP have been described following COVID-19 vaccination (16–31). Here we present a new case of *de novo* iTTP following exposure to the mRNA-based SARS-Cov2-vaccine BNT162b2. In addition, we review previously reported cases in literature and provide an assessment of current evidence.

## Case Report

A 60-year-old man presented to the emergency department with new onset of confusion, nausea, and vomiting (Day 0); 10 days earlier, he had received the second dose of the nucleoside modified mRNA SARS-Cov2 vaccine BNT162b2 (Comirnaty^®^, Pfizer-BioNTech). He reported a 7-day history of progressive exertional thoracic discomfort associated with shortness of breath. His family doctor had diagnosed an *H. pylori* and a urinary tract infection. His medical history was significant for long-standing hypertension, hyperlipidemia, benign prostate hyperplasia, and depression. Notably, one week after the first dose of BNT162b2 he suffered a cerebrovascular ischemic stroke. He was referred to our tertiary care center from a smaller hospital on the following medication: clopidogrel, irbesartan, atorvastatin, escitalopram, esomeprazole, ciprofloxacin, cobalamin, and quercetin.

On arrival, he was in fair general condition (GCS 15; blood pressure 150/80 mmHg; heart rate 70 beats per minute; temperature 34.6°C). During physical examination, the patient experienced a short episode of altered mental state with transient aphasia but had no new focal neurological deficits. The remainder of the examination was unremarkable. A cerebral computed tomography showed residual signs of the prior ischemic stroke but no new findings.

**Figure 1:**
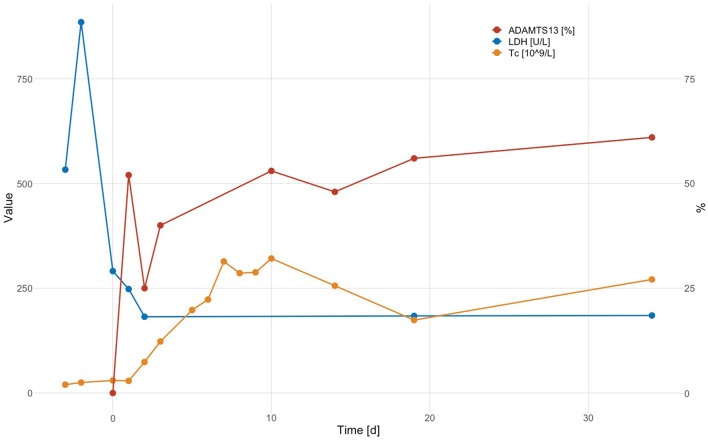
Evolution of platelet count (Tc; × 10^9^/L), LDH level (U/L), and ADAMTS13 enzyme activity (%) (Day 0: day of admission; TPE on Days 1, 2, and 3).

Laboratory results are shown in Table 1. The work-up was notable for microangiopathic hemolytic anemia (hemoglobin 89 g/L, lactate dehydrogenase 885 U/L (normal range <250 U/L), total bilirubin 98 μmol/L (normal range <17 μmol/L), haptoglobin <0.1 g/L, 35% schistocytes on the peripheral blood smear), severe thrombocytopenia (platelet count 25 × 10^9^/L), a normal coagulation profile except for elevated D-dimer levels (1'728 μg/L), and a normal creatinine. High-sensitivity troponin T was transiently elevated while creatine kinase was normal. The ECG showed new flattened anterolateral T waves, but a transthoracic echocardiogram revealed no wall motion abnormalities. Antibodies against platelet factor (PF) 4/heparin complex were negative. A PLASMIC score of 7/7 (32) and a FRENCH score of 2/2 points (33) classified the patient as being at high risk for a severe ADAMTS13 deficiency. A diagnosis of iTTP was confirmed by an ADAMTS13 activity <5% in the presence of a positive anti-ADAMTS13 IgG ELISA result (16.7 AU/mL; normal range <12 AU/mL), the functional ADAMTS13 inhibitor was negative. The patient was treated with daily TPE with replacement of plasma (1.5 × plasma volume on Days 1, 2, and 3) and high-dose oral corticosteroids (1 mg/kg body weight) with a rapid clinical response and normalization of platelet count and LDH after three TPE sessions. Shortly after that, corticosteroids were tapered and stopped. Evolution of platelet count, lactate dehydrogenase level and ADAMTS13 enzyme activity are shown in Figure 1. The patient made a complete clinical remission with an ADAMTS13 activity of 53% on Day 10 and was discharged on Day 11. Hemoglobin levels trailed behind somewhat but had normalized by Day 87. The patient initially was on weekly, then monthly, and is currently on three-monthly follow-ups. During these, ADAMTS13 activity remained stable around the lower limit of normal (44– 61%).

**Table 1:** Laboratory results [Day −10: BNT162b2 vaccine (2^nd^ Dose); Day 0: day of admission].

**Laboratory parameter**	**Reference** **value**	**Day** **–3**	**Day** **–2**	**Day** **0**	**Day** **1**	**Day** **2**	**Day ** **3**	**Day** **5**
Hemoglobin (g/L)	135–168	108	102	89	87	81	85	87
MCV (fL)	80–98	83	84	84	84	86	85	85
Platelet count (x10^9^/L)	150–450	20	25	30	29	74	123	198
Reticulocyte count (G/L)	26.0–78.0			157	146	154	160	193
Indirect bilirubin (μmol/L)	<17	49	48	90	40		6	5
Lactate dehydrogenase (U/L)	<250		533	885	291	248	182	
Haptoglobin (g/L)	0.30-2.00			<0.10	0.51	0.63	0.73	
International normalized ratio	0.7–1.2		1.2	1.18	1.18	1.15	1.15	
Creatinine (μmol/L)	59–104		85	92	84	87	79	80
D-dimer (μg/L)	≤500		3'003	1'728	4'543	1'566	5'543	
C-reactive protein (mg/L)	<5	0.7		4	7	33	6	
Troponin T-hs (ng/L)	<14			32	18	10		12
ADAMTS13 inhibitor (BU/mL)	None			None				
anti-ADAMTS13 IgG (AU/mL)	<12			16.7				
ADAMTS13 enzyme activity (%)	>51			<5	52	63	64	
anti-PF4^*^ antibodies (ELISA)	<0.4			0.147				

* *PF4, platelet factor 4*.

## Immune-Mediated Thrombotic Thrombocytopenic Purpura Following SARS-CoV2 Vaccines

In a literature search in PubMed (performed November 28th, 2021; Purpura, Thrombotic Thrombocytopenic; Thrombotic Thrombocytopenic Purpura; ADAMTS-13 Protein; COVID-19 Vaccine; COVID-19 Vaccine Pfizer-BioNTech; Pfizer Covid-19 Vaccine; COVID-19 Vaccine Johnson & Johnson; COVID-19 Vaccine Moderna; COVID-19 Vaccine AstraZeneca; ChAdOx1 COVID-19 Vaccine; Oxford-AstraZeneca COVID-19 Vaccine), we identified 16 reports describing 20 patients with iTTP following COVID-19 vaccination (16–31). Clinical and laboratory characteristics of all previously reported cases and our case are summarized in Table 2. Including our case (*n* = 21), there were 11 male and 10 female patients; their median age at the time of diagnosis of iTTP following COVID-19 vaccination was 50 years (range 14-84 years). The median time to onset of symptoms was 12 days (range 5–37 days), with 20 patients presenting within 30 days of COVID-19 vaccination (range 5–30 days) (16–27, 29–31) and one patient presenting on day 37 (28). ADAMTS13 activity was <10% or below the detection limit of the assays employed; functional inhibitors or anti-ADAMTS13 antibodies were documented in 18 cases and not reported in the remaining three. Recombinant adenoviral vector-based vaccines were involved in 19% (ChAdOx1-S: 3 cases; Ad26.COV2.S: 1 case), and mRNA-based vaccines were involved in 81% (BNT162b2: 16 cases; mRNA-1273: 1 case), where 10/17 cases occurred after the first and 7/17 cases after the second dose.

**Table 2:** Clinical and laboratory summary of 21 patients including our case.

**References**	**Age (y), sex (M/F)**	**COVID-19 vaccine**	**Symptom onset (days after vaccination)**	**Signs and symptoms**	***De novo*** **/relapse**	**ADAMTS13 enzyme activity**	**Anti-ADAMTS13 antibodies**	**Treatment**	**Clinical course and outcome**
**mRNA-based vaccine**									
Index case	60 y, M	BNT162b2 (2^nd^ Dose)	10	Nausea, vomiting, exertional shortness of breath, thoracic discomfort, jaundice, altered mental state, myocardial ischemia	*De novo*	<5%	16.7 AU/mL^c^ functional inhibitor negative	TPE (3x 1.5 PV), Steroids	Fully recovered. Normalization of platelet count and LDH after the third TPE.
Sissa et al. (16) 04/2021	48 y, F	BNT162b2 (2^nd^ Dose)	6	Ecchymosis on both arms and forearms	Relapse	<3%	88 AU/mL^c^	TPE (7x), Steroids	Fully recovered. Normalization of platelet count and LDH after the third TPE.
Ruhe et al. (17) 06/2021	84 y, F	BNT162b2 (1^st^ Dose)	16	Scattered petechiae, multiple subacute emboli in cranial MRI with partial hemiplegia, acute renal failure	*De novo*	1.6%	82.2 AU/mL^c^	TPE (17x), steroids, RTX	In clinical remission, with ADAMTS13 activity 43–70%. Initial decline in platelet count after seven sessions of TPE.
de Brujin et al. (18) 06/2021	38 y, F	BNT162b2 (1^st^ Dose)	14	Spontaneous bruising and petechiae for several weeks, blurred vision in the left eye for 2 weeks	*De novo*	undetectable^a^	Functional inhibitor 106 BU/mL > 1,000 AU/mL ^c^	TPE (17x 1.5 PV), steroids, RTX (4x), caplacizumab 10 mg for 12 days	In clinical remission. Initial decline in platelet count after six sessions of TPE. ADAMTS13 remained undetectable with a high antibody titer (42 BU^b^).
Pavenski (19) 06/2021	84 y, M	BNT162b2 (1^st^ dose)	7	jaundice, lethargy, myalgia and anorexia the past few days, new infarction	Relapse	undetectable	inhibitor >15^*^	TPE (8x), steroids, RTX (4x)	Fully recovered after 10 days. Initial decline in platelet count after four sessions of TPE. ADAMTS13 activity 47% after 1.5 months.
Maayan et al. (20) 06/2021	40 y, F	BNT162b2 (2^nd^ Dose)	8	Somnolence, low-grade fever, hematuria, petechiae and ecchymosis on lower limbs	*De novo*	Low activity^a^	High antibody titer^c^	TPE (6x), high dose steroids, caplacizumab	In clinical remission, normalization of ADAMTS13 activity.
Maayan et al. (20) 06/2021	28 y, M	BNT162b2 (2^nd^ Dose)	28	Dysarthria lasting 15 min, mild non–specific chest pain several days prior	*De novo*	Low activity^a^	High antibody titer^c^	TPE (5x), high dose steroids, RTX, caplacizumab	In clinical remission, normalization of ADAMTS13 activity.
Maayan et al. (20) 06/2021	31 y, F	BNT162b2 (1^st^ Dose)	13	vaginal bleeding and purpura	Relapse	0%^a^	121 U/mL^c^	TPE (4x), steroids, RTX, caplacizumab	Ten weeks after TPE ADAMTS13 activity still 0%, antibodies present though low, patient continues caplacizumab.
Maayan et al. (20) 06/2021	30 y, M	BNT162b2 (2^nd^ Dose)	8	purpura on limbs	Relapse	Low activity^a^	^c^High antibody titer	TPE (5x), steroids, RTX, caplacizumab	In clinical remission, normal ADAMTS13 activity and antibodies 5 weeks post TPE.
Chamarti et al. (21) 07/2021	80 y, M	BNT162b2 (2^nd^ dose)	12-14	Weakness and malaise jaundice, purpura spots in the buccal mucosa and lower extremities	*De novo*	<2%	inhibitor titer: 182%	TPE, packed RBCs, platelets, steroids, RTX	Fully recovered after 10 days.
Waqar et al. (22) 07/2021	69 y, M	BNT162b2 (2^nd^ dose)	7	Severe fatigue and shortness of breath	*De novo*	<2%	>90 AU/mL^c^	TPE (5x), steroids, RTX (5x)	Fully recovered.
Giuffrida et al. (23) 08/2021	83 y, F	BNT162b2 (1^*st*^ Dose)	7	Fatigue, petechiae, patient refused hospitalization, 7 days later hospitalization, with severe thrombocytopenia and anemia	*De novo*	<10%	40 AU/mL^c^	TPE (2x), steroids, caplacizumab	Died after 2 days of treatment, probably of a sudden cardiovascular event
Giuffrida et al. (23) 08/2021	30 y, F	BNT162b2 (1^*st*^ Dose)	18	Fatigue, diffuse petechiae, and intense headache; whole body CT scan negative	*De novo*	<10%	77. AU/mL^c^	TPE (8x), steroids, caplacizumab	Platelet count normalized on Day 5, discharged on Day 8; caplacizumab for 30 days after stopping TPE, ADAMTS13 activity <10% on Day 30
Kirpalani et al. (24) 08/2021	14 y, F	BNT162b2 (1^*st*^ Dose)	12	Two-day history of fatigue, headache, confusion, bruising	*De novo*	<1%	72 AU/mL^c^	TPE (1.5 PV), steroids, RTX, caplacizumab	Fully recovered. Initial decline in platelet count after five sessions of TPE.
Karabulut et al. (25) 09/2021	48 y, M	mRNA-1273 (1^*st*^ Dose)	5	Transient right-sided weakness, paresthesia, slurred speech lasting approximately 30 min.	Relapse	<3%	functional inhibitor: 6.6 BU/mL	TPE (10x), steroids, FFP, RTX	Fully recovered after 10 days.
Alislambouli et al. (26) 10/2021	61 y, M	BNT162b2 (1^*st*^ Dose)	5	Confusion, fever, emesis headache, dark urine, leg ecchymosis, generalized seizure, new small subdural hematoma 5mm after seizure	*De novo*	<3%	not reported	TPE (12x), steroids, RTX (4x)	Fully recovered. On Day 142 no relapse, ADAMTS13 activity 97%.
Yoshida et al. (27) 11/2021	57 y, M	BNT162b2 (1^*st*^ Dose)	7	Fatigue, loss of appetite, and jaundice	*De novo*	<0.5%	functional inhibitor 1.9 BU/mL^b^	TPE (11x 2 PV), FFP steroids, RTX (4x)	Fully recovered. Initial decline in platelet count after four sessions of TPE.
**Vector-based vaccines**									
Yocum et al. (28) 05/2021	62 y, F	Ad26.COV2.S	37	Altered mental state, GCS 12, scattered petechiae	*De novo*	<12%	not reported	TPE, hemodialysis, packed RBCs, steroids	Fully recovered.
Al-Ahmad et al. (29) 05/2021	37 y, M	ChAdOx1-S	10-15	Progressive dizziness, fatigue, headache, exertional shortness of breath, palpitation	*De novo*	2.6%	positive inhibitory antibodies	TPE (8x 1.5 PV), steroids, RTX (4x)	Fully recovered.
Lee et al. (30) 09/2021	50 y, F	ChAdOx1-S	12	Dysphasia, acute numbness of left upper limb lasting 15 min	*De novo*	0% ^a^	antibodies: >94.93 AU/mL^c^	TPE (14x), steroids, RTX (4x)	Fully recovered. Initial decline in platelet count was noted after six sessions of TPE.
Wang et al. (31) 10/2021	75 y, M	ChAdOx1-S	30	Not reported	*De novo*	0.8%	not reported	TPE (5x) (steroids: not reported)	Thrombocytopenia substantially improved from 9 to 235 G/L, no further details

a *Technozym^®^ ADAMTS-13 activity ELISA; Technoclone*.

b *functional Bethesda method; BU/mL, normal < 0.4 BU/mL*.

c *Technozym^®^ ADAMTS-13 INH ELISA; Technoclone; negative < 12 AU/mL*.

* *Unit not specified by authors*.

*RTX, Rituximab; TPE, plasma exchange therapy; PV, plasma volume; RBCs, red blood cells; FFP, fresh frozen plasma*.

*U/mL, units per milliliter; IU/mL, international units per milliliter; AU/mL, arbitrary units per milliliter; BU/mL, Bethesda units/mL*.

Sixteen patients presented with their first ever iTTP episode, while five patients (24%) had a history of iTTP (16, 19, 20, 25), with a relapse either after the first dose of mRNA-based vaccines in three patients (mRNA-1273: 1 case, BNT162b2: 2 cases) or after the second dose in two patients (both BNT162b2). Pre-vaccination ADAMTS13 activity values were not reported for these five patients.

Treatment included TPE in all patients, with a median of 7.5 (range 2–17) TPE sessions until sustained normalization of platelet counts (number not reported in three cases). Steroids were administered in 20/21 patients (not reported for one case). While four patients (19%) received no additional treatment, weekly rituximab (RTX) or daily caplacizumab was added in 43% (9/21) and 14% (3/21) of patients, respectively, and 5/21 (24%) patients received both. One 83-year-old female patient, who initially had refused hospitalization, died after 2 days of treatment with TPE, corticosteroids, and caplacizumab, which had been initiated seven days after initial presentation, probably due to a cardiovascular event. One other patient, a 31-year-old female, showed a prolonged course with no detectable ADAMTS13 activity 10 weeks after treatment with four TPE sessions, steroids, and RTX, and continued caplacizumab. All other 19 patients fully recovered or were in clinical remission without treatment by the end of the observation period.

We identified no reports in PubMed or the World Health Organization (WHO) pharmacovigilance database VigiBase (https://who-umc.org/vigibase/) of iTTP cases in association with vaccinations with any of the inactivated virus-based vaccines such as CoronaVac^®^ (Sinovac Biotech), or the adenoviral vector-based Sputnik V^®^ (Gam-COVID-Vac, Dr. Reddy's Laboratories) up to November 28th, 2021 (34).

## Discussion

We present an additional case of a first presentation of iTTP in a 60-year-old male 10 days after administration of the second dose of the mRNA-based vaccine BNT162b2 (Comirnaty^®^, Pfizer-BioNTech). A severe ADAMTS13 deficiency (<5% of that in normal plasma) in the presence of a low titer non-inhibitory anti-ADAMTS13 antibody of isotype IgG confirmed the diagnosis of iTTP.

Differential diagnosis of iTTP following SARS-CoV-2 vaccination includes vaccine-induced thrombotic immune thrombocytopenia (VITT) (35–37). The clinical picture of VITT resembles spontaneous autoimmune heparin-induced thrombocytopenia (HIT) and manifests 5-30 days after the first dose of adenoviral-based COVID-19 vaccines. Patients with VITT present typically with moderate-to-severe thrombocytopenia and arterial or venous thrombosis, often at unusual sites such as cerebral sinus veins, splanchnic, and portal veins, but deep venous thrombosis and pulmonary embolism may be present as well (35–37). Patients with acute iTTP episodes usually present with severe thrombocytopenia (<30 G/L), marked hemolysis and diffuse microvascular thrombosis, and although macrovascular thrombosis is possible, it is rare at presentation (1–3). iTTP is characterized by the formation of auto-antibodies against ADAMTS13, which induce a severe ADAMTS13 deficiency through increased clearance of ADAMTS13 or by inhibiting ADAMTS13 function. VITT is characterized by the presence of anti-platelet factor (PF) 4/heparin antibodies (35–37), and ADAMTS13 activity, in case it is determined, is normal or only mildly reduced (36, 37). A combined experimental and computational simulation study has demonstrated an electrostatic interaction with stable complex formation between the ChAdOx1 viral vector and PF4 as a plausible underlying mechanism (38). The initially observed mortality rate of 40-50% has dropped below 25% with increasing awareness of VITT, prompt diagnosis, and adequate treatment (39, 40).

Cardiac involvement with increased troponin is seen in up to 60% of the patients presenting with iTTP (41, 42). In many centers, therefore iTTP patients with acute episodes are initially treated in intensive care units, as was the case in our patient, since arrhythmias and cardiac arrest are important causes of early mortality in iTTP.

Since April 2021, a steadily increasing number of *de novo* cases and relapses of iTTP in temporal association with vaccination against COVID-19 have been reported. Apart from our case, we were able to identify an additional 20 cases in the literature at the end of November 2021. Clinical features of iTTP following COVID-19 vaccination were similar to pre-pandemic iTTP, and excellent response to standard of care treatment was seen in nearly all cases (one death). As survivors of pre-pandemic acute iTTP episodes have a relapse rate of nearly 50%, follow-up with regular ADAMTS13 activity determinations seems warranted in patients who developed iTTP following COVID-19 vaccination.

The observed median onset of symptoms was 12 days (range 5-37 days) after vaccination, with almost all patients presenting within 30 days. The close temporal association lead us to the hypothesis of a causal underlying autoimmune mechanism. As of today, the mechanism that triggers anti-ADAMTS13 antibody formation remains poorly understood. An adverse event following immunization (AEFI) may be caused by the vaccine or may be the result of the immunization process (43). However, AEFIs can also occur after immunization without causal association, and with increasing numbers of administered COVID-19 vaccines, coincidental cases of iTTP following vaccinations may be observed more frequently. Before the COVID-19 pandemic, the annual incidence of first iTTP episodes had been estimated at 1-2.2 cases per million (1–3, 6–9), and seems to have remained stable during the past 2 years. At the end of November 2021, the VigiBase database contained 226 cases of iTTP reported as AEFI related to COVID-19 vaccines. In light of the billions of doses administered, this number and the clinical presentations did not trigger a significant safety signal (34).

Case-based assessment of rare serious AEFIs is critical for identifying potential safety signals that need to be considered for further investigation, and causality assessment is essential for determining the level of certainty, as misconceptions can affect public confidence and adherence to immunization programs (43). To date, the causal link between iTTP and COVID-19 vaccines is weak. Sixty-eight percent (15/21) of previously published cases are classified as an indeterminate causal association to immunization according to WHO guidelines (44). These cases demonstrate a consistent temporal relationship but lack sufficient definitive evidence due to an unknown mechanism explaining a causal link. Our index case is considered unclassifiable as the patient had not been tested for COVID-19, and an acute infection as a triggering factor contributing to iTTP could not be excluded. On admission, no SARS-CoV-2 PCR test was performed, the last negative nasopharyngeal swab was taken 1 month prior, and documentation of seroconversion [presence of anti-Spike and anti-nucleocapsid IgG (45)] was impossible as no pre-TPE plasma samples were available. It is noteworthy that the patient showed no signs of a SARS-CoV-2 infection, and incidence rates were low around the time of admission. Twenty-four percent (5/21) of cases are classified as inconsistent causal association to immunization (coincidental) due to a history of iTTP unrelated to any vaccination. However, relapses of iTTP can be triggered by inflammatory conditions (1–3), and the immunization reaction following a vaccination may act as such. Therefore, ADAMTS13 activity should be determined pre-vaccination in individuals with a history of iTTP episodes.

Regarding the safety of additional doses or boosters of vaccines in patients with episodes of iTTP, no data or recommendations exist. If there is a causal, immune-mediated link between vaccine-induced anti-Spike IgG titers and iTTP, re-exposures would come with an increased risk of additional iTTP episodes. However, the same antibodies are also likely to develop during seroconversion following infection with SARS-CoV-2. If the individual risk profile of patients with suspected vaccine-related iTTP makes additional doses necessary, we suggest shared decision-making, as well as close clinical and laboratory follow-up, including ADAMTS13 activity monitoring in the weeks following vaccination.

In conclusion, iTTP is a rare but life-threatening condition. Given the large scale on which vaccines are currently deployed, it is important to register each one, especially rare adverse reactions. The heterogeneity of iTTP presentations poses a challenge, but maintaining a high degree of suspicion and timely reports to pharmacovigilance authorities will help in increasing the safety of vaccination campaigns.

## Data Availability Statement

The raw data supporting the conclusions of this article will be made available by the authors upon request.

## Ethics Statement

Written informed consent was obtained from the individual(s) for the publication of any potentially identifiable images or data included in this article.

## Author Contributions

FH, VB, NAg, and JK initiated the research. FL, NAg, AW, NAn, and JKH provided patient treatment and follow-up. JKH performed additional laboratory workup. VB, DS, and FH wrote the first draft of the manuscript, which was revised by NA, FH, FL, and JKH. All authors approved the final version of the manuscript.

## Funding

JKH received research funding from the Swiss National Science Foundation (grant 310030-185233). Open access funding was provided by the University of Bern.

## Conflict of Interest

JKH serves on advisory boards of Ablynx/Sanofi (development of Caplacizumab) and Takeda (rADAMST13), honoraria of these activities go to the employer, Insel Gruppe AG. The remaining authors declare that the research was conducted in the absence of any commercial or financial relationships that could be construed as a potential conflict of interest.

## Publisher's Note

All claims expressed in this article are solely those of the authors and do not necessarily represent those of their affiliated organizations, or those of the publisher, the editors and the reviewers. Any product that may be evaluated in this article, or claim that may be made by its manufacturer, is not guaranteed or endorsed by the publisher.
