# Erratum: Immune-mediated thrombotic thrombocytopenic purpura following mRNA-based COVID-19 vaccine BNT162b2: Case report and mini-review of the literature

**DOI:** 10.3389/fmed.2023.1186494

**Published:** 2023-03-27

**Authors:** Frontiers Production Office

**Affiliations:** Frontiers Media SA, Lausanne, Switzerland

**Keywords:** purpura, thrombotic thrombocytopenic, ADAMTS13, mRNA SARS-CoV-2 vaccine, COVID-19 vaccine

An omission to the funding section of the original article was made in error. The following sentence has been added: “Open access funding was provided by the University of Bern.”

The original article has been updated.

